# Puerarin alleviated oxidative stress and ferroptosis during renal fibrosis induced by ischemia/reperfusion injury via TLR4/Nox4 pathway in rats

**DOI:** 10.1590/acb382523

**Published:** 2023-08-04

**Authors:** Jun Jian, Dan Wang, Yufeng Xiong, Jingsong Wang, Qingyuan Zheng, Zhengyu Jiang, Jiacheng Zhong, Song Yang, Lei Wang

**Affiliations:** 1 Renmin Hospital of Wuhan University – Department of Urology – Wuhan, Hubei, China.

**Keywords:** Chronic Kidney Disease, Ferroptosis, Reperfusion Injury, Oxidative Stress

## Abstract

**Purpose:**

To investigate the role of puerarin on renal fibrosis and the underlying mechanism in renal ischemia and reperfusion (I/R) model.

**Methods:**

Rats were intraperitoneally injected with puerarin (50 or 100 mg/kg) per day for one week before renal I/R. The level of renal collagen deposition and interstitial fibrosis were observed by hematoxylin and eosin and Sirius Red staining, and the expression of α-smooth muscle actin (α-SMA) was examined by immunohistochemical staining. The ferroptosis related factors and TLR4/Nox4-pathway-associated proteins were detected by Western blotting.

**Results:**

Puerarin was observed to alleviate renal collagen deposition, interstitial fibrosis and the α-SMA expression induced by I/R. Superoxide dismutase (SOD) activities and glutathione (GSH) level were decreased in I/R and hypoxia/reoxygenation (H/R), whereas malondialdehyde (MDA) and Fe^2+^ level increased. However, puerarin reversed SOD, MDA, GSH and Fe^2+^ level changes induced by I/R and H/R. Besides, Western blot indicated that puerarin inhibited the expression of ferroptosis related factors in a dose-dependent manner, which further demonstrated that puerarin had the effect to attenuate ferroptosis. Moreover, the increased expression of TLR/Nox4-pathway-associated proteins were observed in I/R and H/R group, but puerarin alleviated the elevated TLR/Nox4 expression.

**Conclusions:**

Our results suggested that puerarin inhibited oxidative stress and ferroptosis induced by I/R and, thus, delayed the progression of renal fibrosis, providing a new target for the treatment of renal fibrosis.

## Introduction

Chronic kidney disease (CKD) is a worldwide public health problem with high morbidity and mortality in more than 10% of the global population^1^. Closely associated with renal dysfunction and progression to renal failure, renal interstitial fibrosis was considered to be the ultimate irreversible pathway and the most common pathological change in CKD. It is widely verified that ischemia/reperfusion (I/R) has the capacity to promote kidney injury through aggravating oxidative stress and inflammation, thus leading to renal fibrosis, and the I/R stimuli has become a well-established model in the study of renal fibrosis^2^^,^ 3.

Due to the complex etiology and lack of effective treatment, renal fibrosis had great therapeutic challenges. As the major bioactive ingredient extracted from the herb Pueraria Lobata, puerarin (C_21_H_20_O_9_) is the most important flavonoid in the latter. Previous studies have reported that puerarin might play a vital role in the regulation of cell cycle, autophagy, oxidative stress, inflammation, and insulin resistance-reducing effects^4^^-^ 6. Other ones found that puerarin protected against diabetic nephropathy, acute kidney injury and renal toxic injury^7^^-^ 9 and reversed unilateral ureteral obstruction (UUO) induced renal tubulointerstitial fibrosis in mice^10^. In this study, we aimed to investigate the effects of puerarin on I/R-induced renal fibrosis in rats and to explore the underlying mechanisms.

Ferroptosis is a kind of regulatory cell death caused by iron overload toxicity, lipid peroxidation, and plasma membrane damage^11^. As an essential element of cell metabolism, the level of iron is strictly regulated under physiological conditions to maintain homeostasis^12^. Previous studies revealed that ferroptosis played a vital role in the development of renal fibrosis^13^^,^ 14. Iron deposition leads to secrete fibrinogen in renal tubule epithelial cells, activating neighboring fibroblasts to transform into myofibroblasts or inducing epithelial-mesenchymal transformation (EMT)^15^. It has also been reported that iron deposition and ferroptosis increase significantly when oxidative stress occurs in CKD^16^. Besides, lipid peroxidation and EMT were sharply reduced after ferroptosis inhibited, thus alleviating renal fibrosis^17^. Moreover, puerarin was also reported to participate in nerve and myocardial protection by mitigating oxidative stress, inflammatory factors, and cell ferroptosis^18^^,^ 19. However, it still remains unknown whether puerarin affects ferroptosis during renal fibrosis induced by I/R.

Oxidative stress was regarded as an indispensable part of renal fibrosis. Nicotinamide adenine dinucleotide phosphate (NADPH) oxidase 4 (Nox4), expressed mainly in the kidney, produced reactive oxygen species (ROS) that mediated oxidative stress and promoted fibrosis in CKD^20^. Previous study suggested that ROS-MAPK signaling pathway was involved in fibroblast activation, matrix production, and differentiation into profibrotic myofibroblast phenotype^21^. In addition, oxidative stress was supposed to aggravate renal fibrosis by promoting inflammation, apoptosis, mitochondrial damage, and extracellular matrix (ECM) production^22^^-^ 25. It was also reported that suppression of oxidative stress reduced renal interstitial fibrosis significantly^26^^,^ 27. Moreover, oxidative stress was widely involved in the process of ferroptosis. Previous study found that inhibiting oxidative stress dramatically reduced ferroptosis and inflammation^28^. As an essential antioxidant, puerarin plays a key role in a variety of diseases by alleviating oxidative stress. In this study, we attempted to investigate whether puerarin inhibited the renal fibrosis induced by I/R and the underlying mechanism.

## Methods

### Animal experiments

This study was granted the permission of the Research Ethics Committee of Renmin Hospital of Wuhan University (No.WDRM20191006) and carried out according to guidelines for the Care and Use of Laboratory Animals.

Forty-eight adult male Sprague Dawley rats, aged 10 weeks old and weighing 250-270 g, were provided by the center of experimental animals in the Medical College of Wuhan University (Wuhan, China). Rats were housed under controlled temperature conditions in a 12-hour light-dark cycle and exposed to free access to food and water at the temperature of 25 °C.

The rats were randomly divided into six groups after acclimation as follows, sham group, sham-P1group, sham-P2 group, I/R group, I/R-P1 group, and I/R-P2 group. P1 means the rats were injected with 50 mg/kg of puerarin, and P2 with 100 mg/kg.

According to Wang et al.’s report^29^, 50 and 100 mg/kg doses of puerarin were used for administration. Puerarin injection was dissolved in physiological saline. The rats of P1 and P2 groups were intraperitoneally injected with 50 or 100 mg/kg of puerarin, once a day for seven days, and the sham group was injected with the same volume of physiological saline. On the 7^th^ day, 1 hour after puerarin administration, the rats underwent I/R model, which was performed as previously reported^30^. In brief, the rats were under general anesthesia with pentobarbital sodium (50 mg/kg, i.p.), and then performed midline abdominal incision and right nephrectomy. After exposed the left kidney, the rats were received postoperative analgesia with meloxicam (4mg/kg/d, SC) for three days. Then, noninvasive clips were used for ischemia 45 minutes and then reperfusion 12 weeks.

### Cell cultures

Human kidney-2 (HK-2) cells were provided by the China Center for Type Culture Collection (CCTCC, Wuhan, China). HK-2 cells were cultured in modified Eagle medium (DMEM) (Invitrogen, United States of America), supplemented with 10% heat-inactivated fetal bovine serum under the atmosphere of 5% CO_2_ at 37 °C. In order to establish the *in-vitro* model, three days before hypoxia/reoxygenation (H/R), the cells were treated with puerarin P1 (1uM) and P2 (10uM) once a day respectively, and the control group were treated with the same volume of physiological saline. The H/R process was carried out as described previously^31^. In brief, HK-2 cells were incubated with non-nutrient medium under hypoxic condition (5% CO_2_, 1% O_2_, 94% N_2_) for 12 hours, and then replaced with normal medium and cultured under normoxic condition for 24 hours. The control group was cultured in normal medium under normoxic condition.

### Histopathological staining

Kidney tissue of rats was fixed in 10% paraformaldehyde solution for 24 hours, then embedded with paraffin and prepared into 4-μm sections. The slides were stained with the hematoxylin-eosin (HE) kit (Solarbio, Beijing, China, G1120) and Sirius Red kit (Solarbio, Beijing, China, G1472). The images were photographed under an optical microscope (Olympus Corporation, Japan) at 400× magnification.

### Measurement of SOD, MDA, Fe^2+^and GSH level

According to the manufacturer’s instruction, the activity of superoxide dismutase (SOD) (Xanthine oxidase method) and malondialdehyde (MDA) (Thiobarbituric acid method) in renal tissue lysates or HK-2 cells were measured using the commercial kit (Nanjing Jiancheng company, China). The methods were similar to Kar et al.’s^32^.

The cells or kidney tissues were collected and homogenized with phosphate buffered saline (PBS). The level of normalized iron concentration (Fe^2+^ level) was detected with the Iron Assay Kit (Abcam, China) instruction. The level of glutathione (GSH) was analyzed according to the commercial kit (Nanjing Jiancheng Bioengineering Institute, China).

### Western blot analysis

Kidney tissues and HK-2 cells were lysed with RIPA buffer (Beyotime, Jiangsu, China) containing protease inhibitors to extract total proteins. The total proteins were quantified using the BCA kit (Abcam, Shanghai, China). The lysed sample was separated in sodium dodecyl sulfate (SDS) polyacrylamide gel and then transferred to polyvinylidene difluoride (PVDF) membrane. Subsequently, it was blocked with 5% skimmed milk, followed by overnight incubation at 4 °C with the primary antibodies as follows: anti-4-Hydroxynonenal (4-HNE) (Abcam, ab48506), anti-TLR4 (Abcam, ab8376), anti-NOX4 (Santa Cruz, sc-518092), anti-ACSL4 (Abcam, ab155282), anti-GPX4 (Abcam, ab125066), anti-FSP1 (Abcam, ab197896), anti-α-SMA (Abcam, ab244177) and anti-β-actin (Boster Biological Technology, A01263-4). Then, the membranes were washed and incubated with the secondary antibody for 2 hours. The ImageJ software was used to quantify protein levels.

### Immunofluorescence staining

HK-2 cells were washed twice with PBS, fixed with 4% paraformaldehyde for 15 minutes, and then closed with 5% BSA for 30 minutes. The cells were incubated overnight with anti-α-SMA (Abcam, ab244177) primary antibody at 4 °C. Then, the cells were incubated with fluorescent-conjugated secondary antibodies (Boster Biological Technology, Wuhan, China) at room temperature in the dark for 1 hour. After washing with PBS twice, the nuclei were labeled with 4’,6-diamidino-2-phenylindole (DAPI). Finally, the accumulated optical density was calculated by utilizing ImageJ software.

### Statistical analysis

All data were analyzed with Statistical Package for the Social Sciences (SPSS) 19.0 software (IBM, Armonk, NY, United States of America), and the quantitative data was expressed as the means ± standard error of the mean. Due to the small sample size, the Shapiro-Wilk test was employed to evaluate the normal distribution. As the normal distribution of the data was tested, the comparison between the two groups was conducted using analysis of variance (ANOVA), and Tukey’s multiple comparison test was then applied to determine whether there was statistical significance between the multiple groups. The sample size for this study was calculated using ANOVA test. The outcome suggested that 48 rats (eight in each group) were required to be candidates in this study to meet the significant differences in the groups using 80% performance test (1-β = 0.80), α = 0.05 error (95% confidence interval) with a two-sided hypothesis. P < 0.05 was considered to be statistically significant.

## Results

### Puerarin alleviated renal fibrosis induced by I/R in vivo

**Figure 1:**
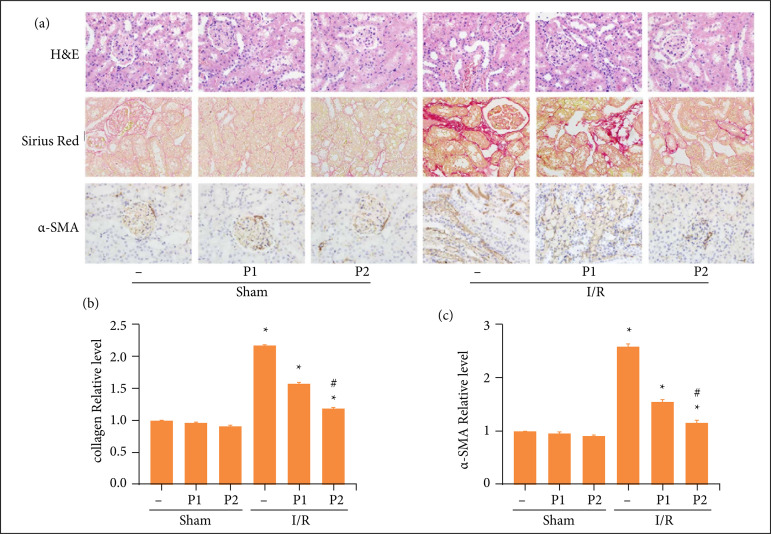
Puerarin alleviated renal fibrosis induced by I/R in vivo. Different concentration of puerarin (50 and 100 mg/kg) was intraperitoneally injected before renal I/R model established, once a day for seven days, and the sham group was injected with the same amount of physiological saline. Then, the rats in I/R group were underwent ischemia 45 minutes and reperfusion 12 weeks: The kidney tissue was collected for staining and photographing in H&E, Sirius Red and immunohistochemical staining (n = 8); Quantitative images of Sirius Red staining; Quantitative images of IHC staining of α-SMA. Three different visual fields were randomly selected for quantitative analysis under the microscope. The staining positive region was extracted by ImageJ software, and the optical density was calculated. Then, the GraphPad prism8 software was used for visualization. Each experiment was repeated at least three times independently.

To explore the effect of puerarin on renal fibrosis, I/R model was established. As shown in Fig. 1, in HE and Sirius Red staining, kidney collagen deposition, and interstitial fibrosis were significantly elevated in I/R group compared with control group. However, puerarin could attenuate the increased collagen deposition and interstitial fibrosis induced by I/R. In accordance with the results of immunohistochemical staining, the expression of α-SMA was dramatically increased in renal I/R, whereas the puerarin reduced its expression in a dose-dependent manner. (Figs. 1a–1c). Therefore, our results suggested that puerarin pretreatment could reduce renal fibrosis induced by I/R in vivo.

H&E: hematoxylin and eosin; α-SMA: α-smooth muscle actin; I/R: ischemia and reperfusion; *P < 0.05 *vs.* sham group; #P < 0.05 *vs.* I/R group.

### Puerarin alleviated ferroptosis and oxidative stress induced by I/R through TLR4/Nox4 pathways in vivo

**Figure 2:**
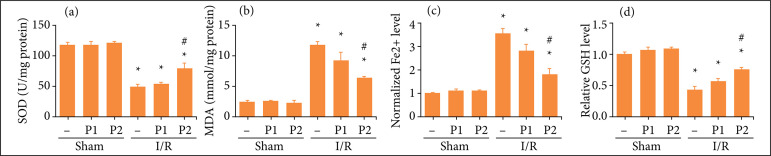
Puerarin reduced oxidative stress and ferrotosis induced by I/R in rats. Different concentration of puerarin (50 and 100 mg/kg) was intraperitoneally injected before renal I/R model established, once a day for seven days, and the sham group was injected with the same amount of physiological saline. Then, the rats in I/R group was underwent ischemia for 45 minutes and reperfusion 12 weeks. **(a)** The measurement of SOD expression in each group; **(b)** the measurement of MDA expression in each group; **(c)** the measurement of GSH expression in each group; **(d)** the measurement of normalized Fe^2+^ level in each group (n = 8). All data were showed as mean ± standard error mean, and two-way analysis of variance was used. Each experiment was repeated at least three times independently.

SOD: superoxide dismutase; MDA: malondialdehyde; GSH: glutathione; I/R: ischemia and reperfusion; *P < 0.05 *vs.* sham group; #P < 0.05 *vs.* I/R group.

Previous studies have demonstrated that UUO led to ROS accumulation, iron deposition and lipid peroxidation, and aggravated renal fibrosis^13^. In this work, we aimed to investigate whether puerarin affected renal fibrosis through regulating ferroptosis and oxidative stress. As shown in Fig. 2, the level of MDA and normalized Fe^2+^ levels were increased (Figs. 2b and 2c) in I/R group, whereas SOD and GSH decreased (Figs. 2a and 2d). However, puerarin pretreatment reversed these changes induced by renal I/R group.

**Figure 3:**
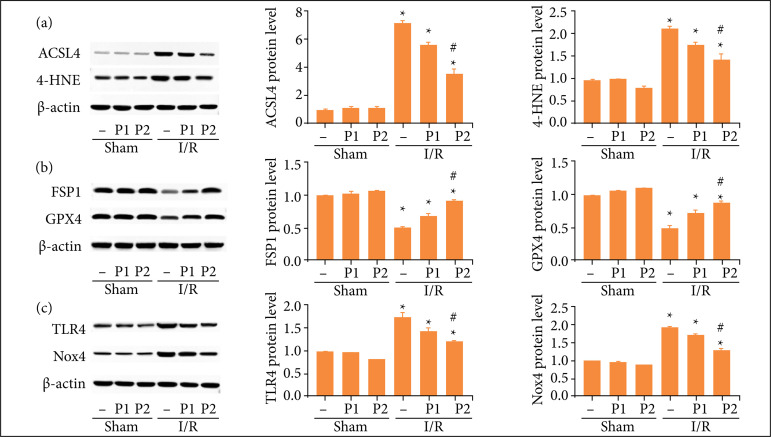
Puerarin alleviated ferroptosis induced by I/R through TLR4/Nox4 pathway in rats. Different concentration of puerarin (50 and 100 mg/kg) was intraperitoneally injected before renal I/R model established, once a day for seven days, and the sham group was injected with the same amount of physiological saline. Then, the rats in I/R group was underwent ischemia 45 minutes and reperfusion 12 weeks. **(a)** Representative Western blot and quantitative images of ASCL4 and 4-HNE expression. **(b)** Representative Western blot and quantitative images of FSP1 and GPX4 expression. **(c)** Representative Western blot and quantitative images of Nox4 and TLR4 expression. β-actin was used for normalization (n = 8). All data were showed as mean ± standard error mean, and two-way analysis of variance was used. Each experiment was repeated at least three times independently.

Western blot revealed that the expression of Acyl-CoA synthetase long-chain family member 4 (ASCL4) and 4-hydroxynonenal (4-HNE) were elevated (Fig. 3a) in I/R group compared with the sham group. However, the expression of ferroptosis suppressor protein 1 (FSP1) and glutathione peroxidase 4 (GPX4) were reduced (Fig. 3b). Moreover, puerarin could reverse these changes induced by I/R in a dose-dependent manner. In order to investigate the possible mechanism, TLR4/Nox4 pathway, closely related to ferroptosis and oxidative stress, was detected in each group. The results showed that TLR4/Nox4 expression was elevated in I/R group, which was reversed by puerarin treatment (Fig. 3c). Therefore, our results demonstrated that puerarin might alleviate ferroptosis and oxidative stress induced by I/R through TLR4/Nox4 pathway in rats.

I/R: ischemia/reperfusion; *P < 0.05, *vs.* sham group; #P < 0.05, *vs.* I/R group.

### Puerarin attenuated α-SMA expression induced by H/R in HK-2 cells

**Figure 4:**
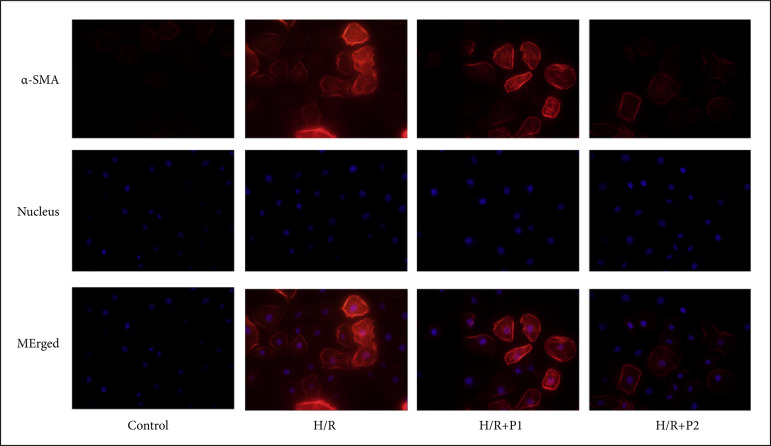
Puerarin reduced the α-SMA expression induced by H/R *in vitro*. Before H/R established, HK-2 cells were treated with puerarin P1 (1uM) or P2 (10uM) for three days, once a day, and the control group were treated with the same amount of physiological saline. Then, the HK-2 cells were cultured under hypoxia conditions for 12 hours and then normoxic conditions for 24 hours. Immunofluorescence staining of α-SMA was performed, and representative images were photographed in HK-2 cells (n = 8). Each experiment was repeated at least three times independently.

Our results suggested that α-SMA expression was significantly increased in H/R group compared with control group, indicating that H/R might facilitate fibrosis in HK-2 cells (Fig. 4). Puerarin pretreatment attenuated the increased α-SMA induced by H/R in a dose-dependent manner (Fig. 4), which indicated that puerarin attenuated renal fibrosis induced by H/R *in vitro*.

α-SMA: α-smooth muscle actin; H/R: hypoxia/reoxygenation.

### Puerarin alleviated ferroptosis and oxidative stress induced by H/R through TLR4/Nox4 pathway in vitro

**Figure 5:**
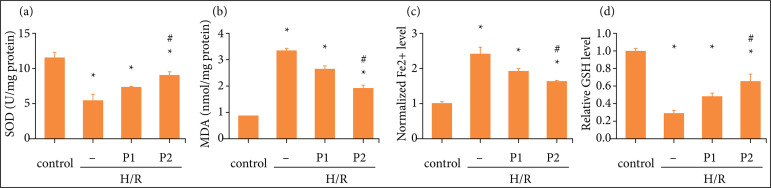
Puerarin decreased oxidative stress and ferrotosis induced by H/R *in vitro*. Before H/R established, HK-2 cells were treated with puerarin P1 (1uM) or P2 (10uM) for three days, once a day, and the control group were treated with the same amount of physiological saline. Then, the HK-2 cells were cultured under hypoxia conditions (5% CO2, 1% O2, 94% N2) for 12 hours and then normoxic conditions for 24 hours. **(a)** The measurement of SOD expression in each group. **(b)** The measurement of MDA expression in each group. **(c)** The measurement of GSH expression in each group. **(d)** The measurement of normalized Fe^2+^ level in each group (n = 8). All data were showed as mean ± standard error mean, and two-way analysis of variance was used. Each experiment was repeated at least three times independently.

Consistent with the *in-vivo* results, the MDA content and normalized Fe^2+^ level were elevated in H/R group compared with control group (Figs. 5b and 5c), while GSH and SOD activity were decreased (Figs. 5a and 5d). Moreover, puerarin pretreatment could reverse these changes induced by H/R in a dose-dependent manner (Fig. 5).

**Figure 6:**
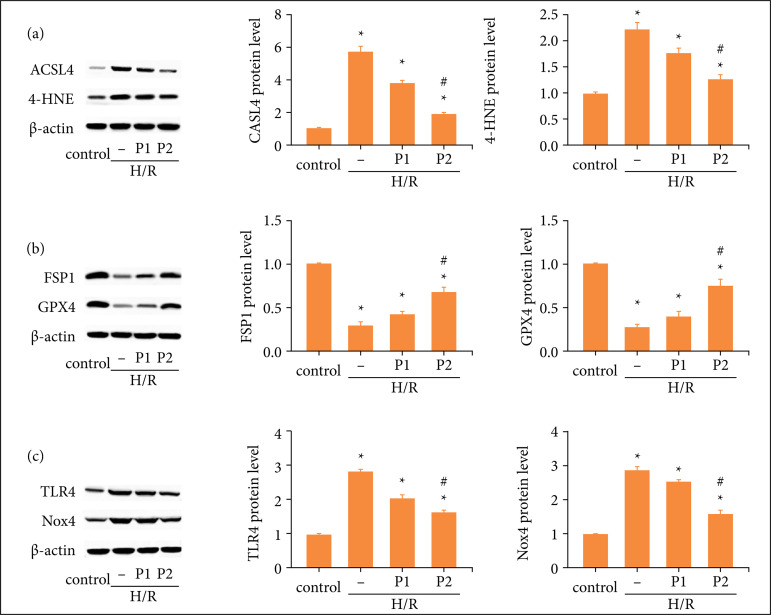
Puerarin alleviated ferroptosis induced by H/R through TLR4/Nox4 pathway *in vitro*. Before H/R established, HK-2 cells were treated with puerarin P1 (1uM) or P2 (10uM) for three days, once a day, and the control group were treated with the same amount of physiological saline. Then, the HK-2 cells were cultured under hypoxia conditions (5% CO2, 1% O2, 94% N2) for 12 hours and then normoxic conditions for 24 hours. **(a)** Representative Western blot and quantitative images of ASCL4 and4-HNE expression in each group. **(b)** Representative Western blot and quantitative images of FSP1 and GPX4 expression in each group. **(c)** Representative Western blot and quantitative images of Nox4 and TLR4 expression in each group (n = 8). All data were showed as mean ± standard error mean, and two-way analysis of variance was used. Each experiment was repeated at least three times independently.

SOD: superoxide dismutase; MDA: malondialdehyde; GSH: glutathione; H/R: hypoxia/reoxygenation; *P < 0.05 *vs.* control group; #P < 0.05 *vs.* H/R group.

Western blot results revealed that the level of ACSL4 and 4-HNE were upregulated, whereas FSP1 and GPX4 expression were downregulated in the HK-2 cells exposed to H/R (Figs. 6a and 6b). Further, puerarin could reverse these changes induced by H/R. In addition, consistent with *in vivo* results, it was found that puerarin inhibited the increased TLR4/Nox4 pathway induced by H/R *in vitro*. In conclusion, the results mentioned suggested that puerarin attenuated ferroptosis and oxidative stress induced by H/R through TLR4/Nox4 pathway *in vitro*.

## Discussion

Renal fibrosis was the ultimate consequence of all CKD, characterized by interstitial fibrosis and glomerulosclerosis, giving rise to progressive and irreversible renal impairment, and eventually leading to end stage of renal disease (ESRD)^33^. Due to the complex etiology of renal fibrosis, the exploration and application of therapeutic targets were urgently needed. In this study, we demonstrated that puerarin might alleviate I/R-induced renal fibrosis in rats for the first time.

Puerarin is an important isoflavone compound extracted from herb *Pueraria lobata* with strong antioxidant activity, which directly or indirectly reinforces energy metabolism, reduces oxidative stress, and plays a vital role in a variety of diseases^34^. According to previous studies, puerarin could reduce diabetic nephropathy and UUO-induced renal fibrosis by alleviating oxidative stress and inflammation^8^^,^ 10. In this study, the results of HE and Sirius Red staining suggested that collagen deposition and interstitial fibrosis were significantly elevated in I/R group compared with sham group. Consistent with previous studies, our study confirmed that puerarin pretreatment dramatically mitigated renal collagen deposition, tubulointerstitial fibrosis and α-SMA expression induced by I/R in a dose-dependent manner. In immunofluorescence staining, the expression of α-SMA in HK-2 cells was increased in H/R group compared with the control group, while puerarin pretreatment reversed this effect. Therefore, our results indicated that puerarin pretreatment could relieve renal fibrosis both *in-vivo* and *in-vitro* models.

Oxidative stress, caused by ROS accumulation, played an important role in renal fibrosis. Nox4 was involved in transforming growth factor (TGF)-β-induced ROS production and the differentiation of myofibroblasts into fibrinous phenotypes^35^. In response to ROS, renal tubular EMT was activated to conduce to renal fibrosis^36^. It was found that suppression of oxidative stress could alleviate renal fibrosis^37^. In previous studies, the level of SOD and MDA were detected subsequently^32^^,^ 38. In this work, it was found that SOD level was decreased in both I/R and H/R model, while the MDA level increased. However, puerarin could reverse the decreased SOD level and increased MDA level induced by I/R and H/R.

Ferroptosis is a programmed cell death caused by ROS accumulation in iron-dependent cells that leads to the breakdown of cell redox homeostasis^12^. It is driven by severe lipid peroxidation and regulated by the lipid repair enzyme GPX4, which relies on the generation of ROS and iron overload^39^. Previous studies have found that ferroptosis inhibitor had the ability to reduce lipid peroxidation and renal fibrosis induced by UUO or hypertension^13^^,^ 17. Others have found that puerarin reduces ferroptosis and thus plays a crucial role in alleviating myocardial injury^19^, lung injury caused by sepsis^40^, and brain injury^18^. However, it still remains unknown whether puerarin regulates ferroptosis in renal fibrosis.

In this study, we measured the GSH and normalized Fe^2+^ level both *in-vivo* and *in*-*vitro* model. Consistent with previous findings, our results showed that GSH expression was reduced during I/R, while the normalized Fe^2+^ level increased dramatically. However, the puerarin pretreatment significantly reversed the changes of GSH and normalized Fe^2+^ level caused by I/R and H/R. Previous study also showed that FSP1 could inhibit ferroptosis effectively, while ASCL4 aggravated ferroptosis^41^^,^ 42. Our results showed that the expression of ACSL4 and 4-HNE was up-regulated, while FSP1 and GPX4 was down-regulated in I/R and H/R groups. Consistent with previous study, our results indicated that puerarin pretreatment reversed the changes of ACSL4, 4-HNE, FSP1 and GPX4 induced by I/R and H/R model, which further demonstrated that puerarin regulated ferroptosis in renal fibrosis.

It also has been found that the silencing of TLR4/Nox4 pathway was correlated to the reduction of oxidative stress and ferroptosis^43^^,^ 44. To further investigate the protection mechanism of puerarin, TLR4/Nox4, closely related with ferroptosis and oxidative stress, was detected in each group. The results showed that TLR4/Nox4 expression increased in I/R and H/R group, which was inhibited by puerarin in a dose-dependent manner. Therefore, our results illustrated that puerarin might alleviate ferroptosis and oxidative stress through TLR4/Nox4 pathway.

## Conclusion

Our research demonstrated that puerarin might alleviate I/R-induced renal fibrosis by attenuating ferroptosis and oxidative stress via TLR4/Nox4 pathway for the first time, providing a new target for the therapy of renal fibrosis.

## Data Availability

The data will be available upon request.
